# Acute and chronic cardiac radio frequency ablation lesion visualisation using magnetic resonance imaging

**DOI:** 10.1186/1532-429X-11-S1-O83

**Published:** 2009-01-28

**Authors:** Benjamin R Knowles, Dennis Caulfield, Michael Cooklin, Aldo Rinaldi, Reza Razavi, Tobias Schaeffter, Kawal S Rhode

**Affiliations:** 1 King's College London, London, UK; 2 Guy's and St Thomas' NHS Foundation Trust, London, UK

**Keywords:** Visualisation Method, Radio Frequency Ablation, Late Enhancement, Ablation Lesion, Healthy Myocardium

## Introduction

The use of electro-anatomical mapping systems (EAMS) for catheter guidance and ablation point recording is widespread for the treatment of atrial arrhythmias using radio frequency ablation (RFA). Also, the evolution of RFA lesions over time may be an important factor in the reoccurrence of arrhythmias. In this study, we use late enhancement (LE) magnetic resonance imaging (MRI) as a tool to measure RFA lesions and present a novel visualisation method to represent this information in an intuitive way. This allows the validation of the in-vivo accuracy of EAMS and the examination of the evolution of RFA lesions from the acute to the chronic timescales.

## Methods

Six patients with either atrial fibrillation (AF, 5 cases) or flutter (AFL, 1 case) underwent RFA. Prior to the procedure, each patient underwent a MRI examination that included administration of a double dose of Gd-DTPA contrast agent followed by MR angiography (MRA) and a T_2_-prepared balanced-SSFP (bSSFP) sequence. Approximately 20 minutes after contrast administration, a free-breathing, cardiac triggered, 3D LE scan was performed. The scan was inversion recovery-prepared with a resolution of 1.3 × 1.3 × 2 mm^3^ and TR/TE/á of 6.2 ms/3.0 ms/30°. Signal was acquired using Turbo Field Echo (TFE) with a 100 ms window and a low-high K-space ordering. Inversion time was determined from a Look-Locker scan. All scans were performed using a 1.5 T Philips Achieva MR scanner. During the procedure, ablation points from various EAMS (NavX, CARTO, XMR-EAMS [1]) were recorded. Post ablation, the patient returned to the MR scanner for a further examination including the bSSFP and LE scans. Approximately 6 weeks later, the patient returned for follow-up MR imaging. Offline, an atrial surface model was generated from the MRA scan, and was transformed into the coordinate system of each of the LE images. Integration of the LE image along the normal vector at each of the surface vertices was performed. This integral was used to colour code the surface model. Areas of LE were defined by integral values higher than the mean of the healthy myocardium plus three standard deviations. EAMS data from NavX and CARTO were exported. The EAMS-derived and the MR-derived cardiac surfaces were registered using a landmark-based registration. Using this registration, the EAMS lesions were transformed onto the MR-derived cardiac surface and repositioned at the nearest vertex.

## Results

**Figure 1:**
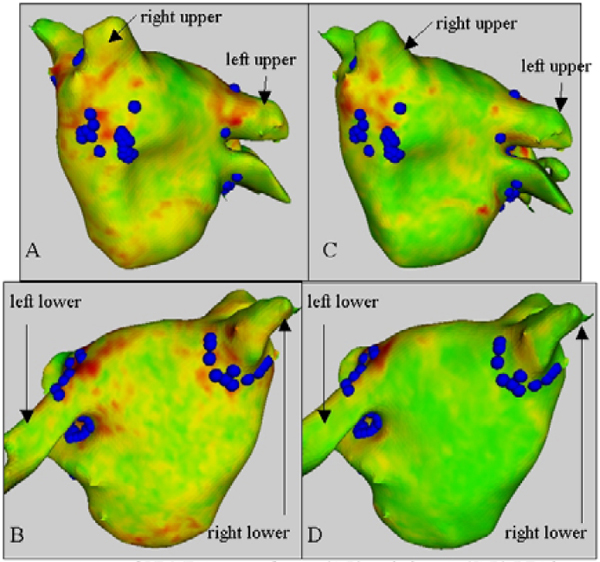
**3D representation of LE MR images of acute (A-B) and chronic (C-D) RFA lesions**. Overlaid in blue are the ablation points acquired with NavX. It can be seen in the chronic image that an area of enhancement is no longer apparent around the right lower PV.

Enhancement was present in all acute and chronic LE images. However, on inspection of the lesion patterns in three dimensions it could be ascertained that from the five patients undergoing PV isolation, one had isolation of both the left and right PVs, three had isolation of the left PVs, and one had isolation of neither. On comparing with the points acquired from NavX, on average 50% of the points were found in the MR-defined area of enhancement. In the patient after AFL ablation, a line of enhancement between the inferior vena cava and the tricuspid valve was visible, and 100% of ablation points acquired from the XMR-EAMS were found in an area of LE. The change in scarring between acute and chronic lesions was found to be considerable in all cases. Enhancing areas were either greatly reduced or non-existent, as shown in figure 1.

## Conclusion

We have presented a technique based on LE MRI coupled to a novel visualisation method to measure RFA lesions. This technique has been applied to six patients undergoing RFA and used to assess the accuracy of EAMS and to monitor the evolution of lesions over time. We envisage that such an approach will have potential benefit in understanding the causes of arrhythmia reoccurrence and also in the guidance of redo ablations.
